# Overview of the cellular and immune mechanisms involved in acute pancreatitis: In search of new prognosis biomarkers

**DOI:** 10.1017/erm.2024.40

**Published:** 2025-01-06

**Authors:** Alexandra Mititelu, Alina Grama, Marius-Cosmin Colceriu, Tudor L. Pop

**Affiliations:** 1 2^nd^ Pediatric Discipline, Department of Mother and Child, Iuliu Hatieganu University of Medicine and Pharmacy, Cluj-Napoca, Romania; 2 2^nd^ Pediatric Clinic, Emergency Clinical Hospital for Children, Cluj-Napoca, Romania

**Keywords:** acute pancreatitis, biomarkers, immune system, pathogenesis, prognostic, severity

## Abstract

Acute pancreatitis (AP) is an acute-onset gastrointestinal disease characterized by a significant inflammation of the pancreas. Most of the time, AP does not leave substantial changes in the pancreas after the resolution of the symptoms but the severe forms are associated with local or systemic complications. The pathogenesis of AP has long been investigated and, lately, the importance of intracellular mechanisms and the immune system has been described. The initial modifications in AP take place in the acinar cell. There are multiple mechanisms by which cellular homeostasis is impaired, one of the most important being calcium overload. Necrotic pancreatic cells initiate the inflammatory response by secreting inflammatory mediators and attracting immune cells. From this point on, the inflammation is sustained by the involvement of innate and adaptive immune systems. Multiple studies have demonstrated the importance of the first 48 h for identifying patients at risk for developing severe forms. For this reason, there is a need to find new, easy-to-use and reliable markers for accurate predictions of these forms. This review provides an overview of the main pathogenetic mechanisms involved in AP development and the most promising biomarkers for severity stratification.

## Introduction

Acute pancreatitis (AP) is an important cause of hospitalization and a burden for the healthcare system through the resources needed for the management and follow-up of these patients (Refs 1, 2). It is characterized by the acute inflammation of the pancreas that can evolve into multiple organ failure and, consequently, have significant morbidity and mortality (Refs 3, 4). In recent years, a progressive increase in the incidence of AP has been observed, both in the adult and paediatric populations, up to 74 cases per 100,000 and 13 cases per 100,000, respectively (Refs 5, 6). The reason for this increase is not understood but it seems to be determined by increased recognition and environmental and lifestyle changes (Ref. 6).

The diagnosis of AP is currently established according to the revised Atlanta classification and it requires the fulfilment of two of the following three criteria: abdominal pain characteristic for AP, serum lipase or amylase values over three times the upper normal limit and imaging findings characteristic for AP on abdominal ultrasound, computed tomography or magnetic resonance imaging (Ref. 7).

The revised Atlanta classification also establishes the severity of the AP, which recognizes three AP forms: mild AP, in which local or systemic complications are absent; moderate AP, including cases with local complications like pancreatic/peripancreatic collections and/or organ dysfunction that resolves in <48 h; and severe forms including patients with organ dysfunction that persists for more than 48 h, many of them developing multiple organ dysfunction syndrome (MODS) (Ref. 7).

AP has different aetiology between adults and children, but regardless of the triggering factor, the pathogenetic mechanisms are the same (Refs 8, 9). These mechanisms have long been investigated but many aspects remain poorly understood. The initial modification appears in the acinar cell. Several changes impair cellular homeostasis, one of the most studied being premature trypsinogen activation. However, in recent years, other mechanisms like intracellular calcium overload or transcription factor activation have been demonstrated to be very important (Refs 10, 11). Destructed acinar cells initiate the inflammatory response by attracting immune cells, such as neutrophils and macrophages. Recently, the involvement of the adaptive immune system has been demonstrated as an essential player in the augmentation and diminution of inflammation (Refs 11, 12). Numerous unanswered questions remain regarding the immune dysregulation that differentiates between a local limited form and a severe one characterized by multiorgan involvement.

As stated before, AP is a significant cause of mortality, especially the severe form in which it reaches up to 50% (Ref. 7). For this reason, several scoring systems have been studied for the prognosis of severe forms, but none showed reliable predictive values and are difficult to apply (Ref. 4). The problem is even more significant if we look at paediatric patients, for which these scores cannot be used (Ref. 9). These gaps in understanding and managing AP have led to the need to identify new, more reliable makers for the prognosis values and the possible therapeutic purpose.

This review discusses the major mechanisms involved in initiating and progressing AP, intracellular changes and immune system involvement, as well as the most promising markers that could serve to predict the severe forms and even as therapeutic targets.

## Pathogenetic mechanisms of acute pancreatitis

### Cellular injury

**Figure 1.**
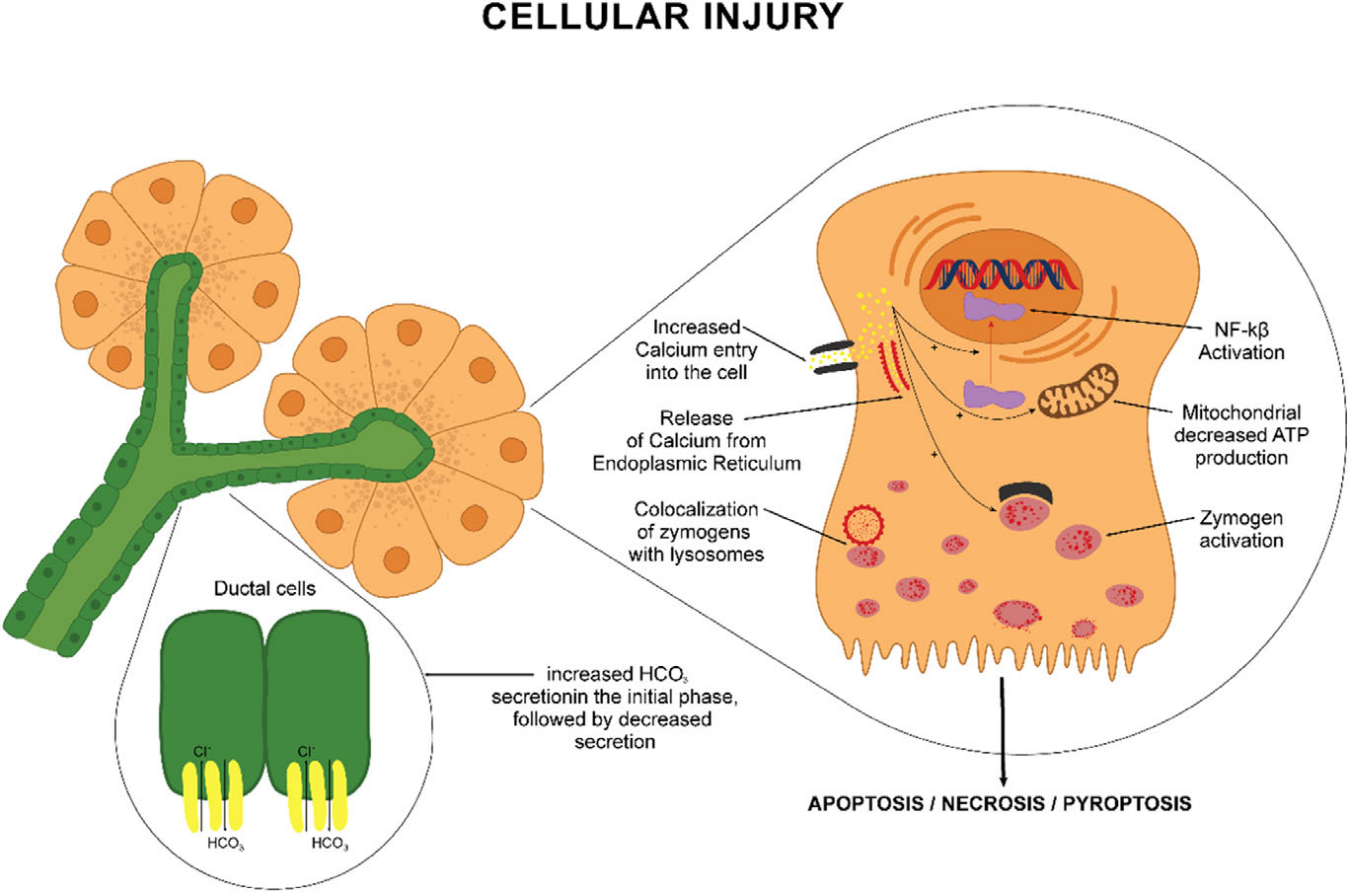
Multiple cellular mechanisms determine apoptosis/necrosis of the acinar cell. Calcium overload is the central mechanism that activates a cascade of events. The intracellular calcium comes from increased ER release and the extracellular space influx. This calcium overload impaired cellular homeostasis by several mechanisms: activation of the nuclear factor κB (NF-κB) pathway and cytokine synthesis; mitochondrial dysfunction and decreased ATP production followed by cellular death; trypsinogen activation and autodigestion followed by cellular destruction; colocalization of zymogens and lysosomes, ductal cell dysfunction. All these processes determine the destruction of acinar cells through necrosis, apoptosis or pyroptosis.

For many years, the central AP mechanism was considered the premature activation of trypsin and autodigestion of the acinar cells. However, it is now clear that several mechanisms produce acinar cell dysfunction and inflammation initiation (Ref. 11). The main mechanisms involved are premature activation of trypsin, colocalization of zymogen granules with lysosomes, calcium signalling and activation of the transcription factor nuclear factor-κB (NF-κB) (Ref. 12). Figure 1 show the main cellular mechanisms.

#### Premature trypsinogen activation

The central role of trypsin activation in the pathogenesis of AP has long been demonstrated, but the mechanisms by which trypsinogen gets activated are not entirely understood. The most substantial evidence supporting the importance of trypsin activation comes from the hereditary forms of pancreatitis. Mutations in the genes encoding cationic trypsinogen (*PRSS1*) determine an increased susceptibility to acute and chronic pancreatitis by making molecules more susceptible to activation. Similarly, the mutations in the gene encoding the serine protease inhibitor (*SPINK1*) cause an autosomal recessive susceptibility by secreting an inactive molecule (Refs 13, 14). Additional support for this hypothesis comes from studies with experimental models of pancreatitis. In a study that used knockout mice lacking trypsinogen isoform-7, there was no intra-acinar trypsinogen activation, but there was evidence of NF-κB activation, and systemic inflammation was not altered (Refs 13, 15). These findings concluded that trypsinogen activation could be an important factor in initiating acinar injury and inflammation cascade from AP but it is not mandatory for the appearance of the disease; other mechanisms determine cellular damage and inflammatory response (Refs 13, 14).

#### Colocalization of zymogens with lysosomes

Various pancreatic injuries like trauma, ductal obstruction or alcohol can determine the fusion between the lysosome and zymogen granules inside the acinar cell. This process is called colocalization, and in these vacuoles, cathepsin B, a lysosomal enzyme, determines the activation of trypsinogen to trypsin. After the enzymes are released from the vacuoles, trypsin causes autodigestion, but cathepsin B also plays an important role in cellular destruction. Experimental studies demonstrated that small amounts of cytosol cathepsin B initiate cell death by apoptosis. In contrast, large amounts shift the pathway towards necrosis, thus being an important molecule in the initial phases of AP (Refs 13, 16–18).

#### Calcium signalling

Usually, intracellular calcium handling is an important mechanism for the function of the acinar cell. Calcium release from endoplasmic reticulum (ER) is associated with zymogen exocytosis in the apical pole and stimulates ATP production in the mitochondria. This increase in cytosolic levels of calcium is transient and, in physiological conditions, the levels rapidly decreased by two ATP-dependent calcium channels: the smooth-ER calcium channel, which moves back the calcium into the ER (SERCA- ssarcoplasmic/endoplasmic reticulum Ca^2+^ ATPase), and the membrane calcium channel, which removes calcium from the cell (PMCA - plasma membrane Ca^2+^ ATPase) (Refs 16, 19). Alcohol and bile acids have been demonstrated to disrupt intracellular calcium homeostasis by increasing calcium levels from the ER store and, afterwards, entering extracellular calcium (Refs 12, 16, 20). Furthermore, other aetiologies have been associated with the calcium signalling mechanism, like medication (asparaginase) and post-endoscopic retrograde cholangiopancreatography (ERCP) pancreatitis, making it one of the most important pathways for cellular injury (Refs 21, 22). The build-up of calcium levels in the acinar cell triggers several important mechanisms that eventually lead to cellular death: trypsinogen auto-activation, vacuole formation, cytoskeletal damage, NF-κB activation and maybe, most importantly, mitochondrial dysfunction. The calcium overload results in a loss of membrane potential to produce ATP, accentuating calcium accumulation by decreasing the function of ATP-dependent channels SERCA and PMCA (Refs 12, 16, 23–25). All this evidence suggests that increased intracellular calcium has a central role in the initiating phases of AP. This observation led to the search for potential therapeutic targets that could prevent calcium overload, some of which had encouraged initial results (Ref. 21).

#### Autophagy and endoplasmic reticulum stress

Autophagy is an essential cytoprotective process through which cytoplasmatic proteins and organelles are degraded and recycled. Targeted molecules are entrapped into autophagic vacuoles, which merge with lysosomes and are digested by proteolytic enzymes. In AP, autophagy is impaired, resulting in ER stress, premature trypsinogen activation and mitochondrial dysfunction (Refs 14, 16).

ER stress means the accumulation of misfolded proteins inside the ER that happens when the normal function of the ER is overwhelmed and protein elimination is impaired. In AP, ER stress is determined by an increased demand for protein production and defects in recycling proteins (Refs 16, 24, 26).

#### Ductal cell dysfunction

The most investigated association between ductal cell involvement in AP is with the cystic fibrosis transmembrane regulator mutations that cause increased susceptibility to developing the disease by reduced secretion (Ref. 16). Secretion of bicarbonate from the ductal cells is an essential mechanism for the neutralization of acidic content produced by acinar cells. It is regulated by pH-sensing mechanisms and membrane transporters (Refs 27, 28). Recent studies regarding ductal cell function in AP revealed an increased secretion in the initial phase, followed by a reduction afterwards, which seems to be a protective mechanism intended to wash out the toxins (Ref. 29). Another mechanism of ductal damage is increased pressure caused by gallstone obstruction or contrast injection during ERCP, which can activate the pathological calcium signalling pathway in the acinar cells. Other modifications are fluid stasis, which promotes premature enzyme activation, intraductal acidification and ductal cell exposure to bile acids (Ref. 16).

#### NF-κB activation

NF-κB is a transcription factor that regulates several genes involved in immune and inflammatory response by increasing the production of cytokines and chemokines and the activation of innate immune cells and inflammatory lymphocyte T cells (Refs 30, 31). The involvement of NF-κB in acute pancreatitis comes from experimental studies that demonstrated reduced severity of pancreatitis in a model with NF-κB deletion. The exact activation mechanisms are not fully understood but evidence suggests calcium signalling and the production of reactive oxygen species are the most important ones. NF-κB activation in AP determines cytokine synthesis and initiation of local inflammatory response through transcriptional induction of proinflammatory cytokines, chemokines and other inflammatory mediators like inflammasomes (Refs 30, 32).

#### MicroRNAs

MicroRNAs (miRNAs) are single-stranded nucleotides that belong to the non-coding RNA class. These nucleotides play an important role in regulating gene expression and, thus, are involved in cell growth, development and death (Refs 33, 34). The biogenesis of miRNAs is a complex process. Typically, they bind to a complementary sequence in the 3′-untranslated region of messenger RNA (mRNA), causing mRNA silencing or degradation. A single miRNA can regulate the expression of hundreds of genes (Ref. 35). Recently, there has been increasing evidence of the functional role of miRNAs in acinar cell injury, inflammatory response and distal organ involvement (Ref. 34). The first evidence to support miRNAs in AP came from studies on animal models that revealed significant upregulation of miR-135a and miR-22 in the pancreatic tissue (Ref. 36). Afterwards, many miRNAs, like miR-29a and miR-21-5p, seem to be upregulated in pancreatic acinar cells during AP and induce cellular death through apoptosis, necrosis or pyroptosis (Refs 37, 38). Furthermore, miRNAs participate in the immune cell regulation in the progression of AP by interacting and upregulating macrophages, mononuclear cells and even T lymphocytes (Refs 39–41). miRNAs seem to be involved in AP-associated lung injury, miR-21 being upregulated in lung tissue during severe AP (Ref. 42). Due to this evidence and the good stability of these markers, miRNAs could be considered as possible predicting markers for severity stratification (Refs 38, 43).

#### Apoptosis and necrosis

The final result of all the above-described changes in the acinar cell is cellular death, which is produced either by apoptosis, necrosis or pyroptosis. While apoptosis is a form of programmed cellular death mediated by caspases, necrosis is a process of self-destruction caused by internal or external factors (Ref. 44). In AP, necrosis seems to be the main type of pancreatic acinar cell death and the release of damage-associated molecular pattern molecules (DAMPs) from necrosis is implicated in the inflammatory response. The mechanism leading to one or another form of cellular death is still unknown but may be a crucial factor for disease progression (Refs 23, 44).

#### Pyroptosis

Pyroptosis is a form of cellular death that involves the death of immune cells mediated by inflammasomes secondary to an immune stimulus (Ref. 44). This type of cellular death has some features similar to apoptosis and necrosis, like DNA fragmentation and cytoplasmic swelling. Unlike apoptosis, pyroptosis has a proinflammatory effect due to the rapid plasma membrane permeabilization and early membrane rupture, as well as the secretion of proinflammatory cytokines (Refs 44, 45). Pyroptosis progression is a caspase-dependent process. Both the canonical pathway, which involves caspase-1 activation, as well as the noncanonical pathway, involving caspase-4, -5 and -11, can initiate pyroptosis (Ref. 46). There is increasing evidence that shows the importance of the inflammasomes and, subsequently, pyroptosis in the progression of inflammation in AP. Therefore, pyroptosis-related molecules could be an independent prognostic marker for AP severity but pyroptosis could also provide a therapeutic target for severe forms in the future (Ref. 47).

The pathogenetic modifications are the same after initiating the inflammatory process, regardless of the aetiology. Nevertheless, the trigger may differ between the different aetiologies. The main aetiologies and the triggering mechanisms are summarized in Table 1.

**Table 1.** The main mechanisms for the initiation of AP associated with the most important causes

Main aetiologies of AP		Initiating pathogenic mechanisms
Hereditary	SPINK1	A form of trypsinogen more susceptible to auto-activation (Ref. 48)
	PRSS1	Reduced inhibitor expression and compromised trypsin inhibition (Ref. 48)
	CTRC	Reduced secretion or activity of chymotrypsin C and thus impaired protective trypsinogen degradation (Ref. 48)
	CFTR	Decreased ductal cell secretion (Ref. 48)
Biliary (bile acids)		Increased calcium levels from ER stores Premature trypsinogen activation NF-κB activation (Ref. 21)
Alcohol		Increased intracellular calcium Ductal cell dysfunction by decreased HCO_3_^−^ secretion (Ref. 21)
Infections		Direct cytotoxic effect on the acinar cells Activation of NF-κB pathway (Ref. 49)
Medication	Asparaginase	Increased calcium levels Mitochondrial dysfunction leading to necrosis (Ref. 21)
Post-ERCP		Increased pressure in the pancreatic ducts Premature trypsinogen activation (Refs 21, 50)
Metabolic	Hypercalcaemia	Increased intracellular calcium levels Activation of NF-κB pathway (Ref. 51)
	Hypertriglyceridemia	Ischaemia due to increased serum viscosity Acinar cellular death through necrosis (Ref. 52)

*Note:* AP, acute pancreatitis; CFTR, cystic fibrosis transmembrane regulator; ERCP, endoscopic retrograde cholangiopancreatography; NF-κB, nuclear factor-κB.

### Innate immune response

The innate immune system is the first line of defence against pathogens. It is a nonspecific but very rapid protection mechanism without immunological memory. There are some molecules released by the pathogens that are critical in the initialization of the innate immune response: pathogen-associated molecular patterns (PAMPs), lipopolysaccharides or double-stranded ribonucleic acid (RNA). In the innate immune response, many types of cells are implicated: phagocytes (neutrophils and macrophages), dendritic cells (DCs), mast cells and natural killer (NK) cells (Refs 53, 54).

**Figure 2.**
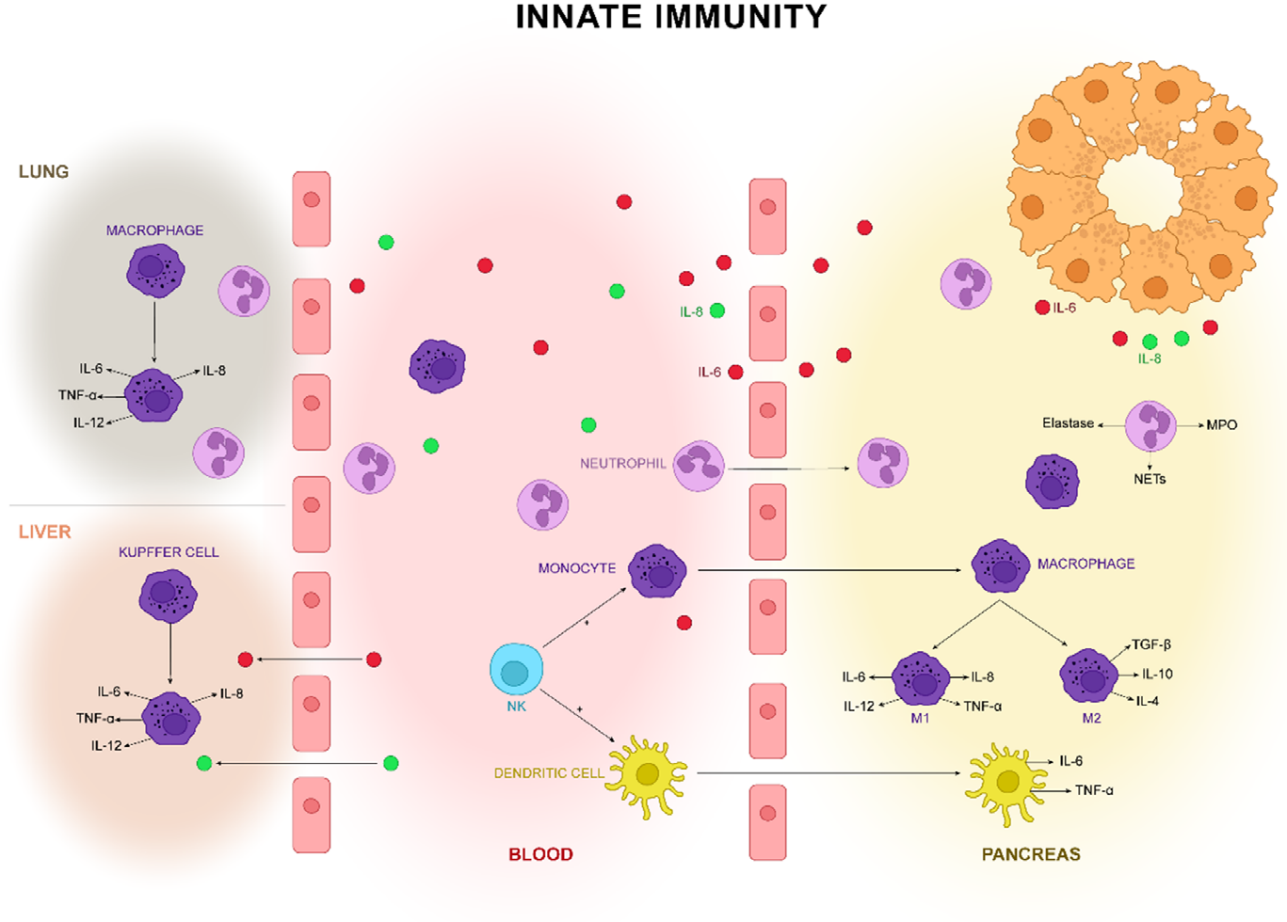
Necrotic acinar cells release proinflammatory cytokines and chemokines. They are crucial in attracting immune cells to the pancreas: neutrophils, macrophages, dendritic cells and natural killer (NK) cells. The neutrophils are the first ones that migrate to the pancreas and determine augmentation of inflammation augmentation through the release of elastase, myeloperoxidase (MPO) and neutrophil extracellular traps (NETs). Proinflammatory cytokines cause activation of resident macrophage as well as migration of blood monocytes. In the pancreas, monocytes differentiate into M1 (proinflammatory secreting macrophages) and M2 (anti-inflammatory secreting macrophages). The M1/M2 ratio imbalance is an important step in the augmentation of inflammation in acute pancreatitis (AP). Nevertheless, the circulating proinflammatory cells activate the resident macrophages from the liver and lung and determine the magnitude of systemic complications. Dendritic cells also migrate in the pancreas tissue and are involved in the secretion of proinflammatory molecules. The NK cells have a bivalent role, either stimulating macrophages and dendritic cell activation or destroying them by cytotoxic effect.

In AP, damaged acinar cells release cytokines, chemokines and DAMPs, which determine the rapid recruitment of immune cells in the pancreas. The main cytokines released in the first stages of inflammation or infection are tumour necrosis factor (TNF), interleukin 1 (IL-1) and IL-6 (Refs 54, 55). Figure 2 show the main innate cells involved in AP.

#### Neutrophils

Neutrophils are the predominant leukocyte subpopulation in the blood, derived from bone marrow haematopoietic stem cells (Ref. 56). It has been demonstrated that neutrophils are an important factor involved not only in infections but also in sterile inflammation (Ref. 57). Thus, neutrophils are one of the first cells recruited in the pancreas by DAMPs or by the chemokines and leukotriene released by the surrounding tissue to respond to DAMPs production. DAMPs include histone, DNA, IL-1α and many others. From the chemokine family, IL-8 (CXCL8) has the most important role in neutrophil chemotaxis and has been demonstrated to be an essential predictive marker for severity (Refs 57–59).

The first step of the recruitment is the interaction with the endothelium, which is enhanced by adhesion molecules from the selectin and integrin family, like intercellular adhesion molecule 1 (ICAM1). Another essential molecule for neutrophil migration is metalloproteinase, especially matrix metalloproteinase-9 (MMP-9), which helps by degrading some components of the basement membrane so the neutrophils can reach the pancreatic tissue (Refs. 59–61). Once infiltrated in the pancreas, neutrophils perform their functions by phagocytosis, degranulation and neutrophil extracellular trap (NET) production. The most important molecules released by neutrophils are elastase, reactive oxygen species (ROS) and myeloperoxidase, which are involved in the activation of trypsinogen, cytokine release and inflammation enhancement (Refs 57, 59).

NETs are another way for neutrophils to fight bacterial infections, but they have also been involved in sterile inflammation. NETs contain DNA, histones and cytosolic proteins and they are usually formed once neutrophils are dead (Refs 56, 59).

Neutrophils are also involved in the systemic complications that are associated with severe AP, especially with acute lung injury (ALI). The first phase of ALI is the exudative phase, which involves microvascular damage and endothelial barrier dysfunction induced by inflammatory mediators. These changes determine neutrophil recruitment in the pulmonary interstitium and alveolar space that become activated and produce an increased inflammatory response, similar to the effect on the pancreas (Ref. 62).

Although neutrophils play a significant role in exacerbating inflammation, they also contribute to tissue repair. This is achieved by the phagocytosis of cellular debris, but most importantly, by the apoptosis of neutrophils cleared by macrophages. The clearance process activates the secretion of anti-inflammatory cytokines like tissue-repairing cytokines transforming growth factor-β (TGF-β) and IL-10 (Refs 59, 63).

#### Macrophages

Macrophages are one of the most important cells involved in the evolution of AP. Pancreatic macrophages include tissue-resident macrophages and migrating macrophages (circulating monocytes) (Ref. 64). DAMPs, proinflammatory mediators released by pancreatic injured tissue, necrotic cells like monocyte chemoattractant protein-1 (MCP-1) and TNF-α induce resident macrophage activation and monocyte migration (Ref. 56). After macrophage recruitment, the predominant cytokine environment determines the orientation of macrophage polarization, meaning that there are two distinct subsets: M1, classically activated, and M2, alternatively activated. M1 macrophages induce a Th1-type response by releasing cytokines and chemokines, accentuating the proinflammatory response (Refs 64, 65). Mosser proposed an alternative classification of macrophages based on three homeostatic activities, which are host defence, wound healing and immune regulation. Furthermore, tumour-associated macrophages have also been identified as a separate group extensively studied in the last few years (Refs 66, 67). The process of macrophage differentiation is called plasticity and describes the ability of cells to respond to different microenvironmental influences by displaying diverse functional phenotypes. Unlike other cells, which lose their heterogeneity during maturation, macrophages retain their plasticity and transform according to environmental signals (Refs 67, 68).

The severity of the pancreatitis episode is correlated with the degree of macrophage activation and the M1 to M2 ratio imbalance; M1 predominance is associated with more severe forms (Refs 57, 64, 69). The central role of pancreatic macrophages in the evolution of AP is due to the involvement, besides the cytokine secretion, in trypsinogen activation, NF-κB activation and adaptive immune response (Ref. 64).

In addition to pancreatic macrophages, peritoneal, liver and alveolar macrophages play an important role in the evolution of AP. The proinflammatory cytokines and enzymes are released in the bloodstream and the peripancreatic area, inducing the activation of peritoneal macrophages to the M1 subtype. The activated peritoneal macrophage can synthesize chemokines and cytokines like IL-1β, TNF-α and IL-8, aggravating the inflammatory response (Refs 64, 70).

Kupffer cells are the resident macrophages of the liver and become activated in the evolution of pancreatitis by the cytokines and enzymes that reach the liver through the portal vein. They influence the magnitude of inflammatory systemic response and pulmonary involvement (Refs 64, 71).

The alveolar macrophages are activated by the proinflammatory cytokines released by the pancreas and the Kupffer cells, which reach the lung through the blood. The resident pulmonary macrophages determine the severity of pulmonary involvement by cytokine and ROS release (Refs 64, 72, 73).

#### DCs

DCs link innate and adaptative immune systems by functioning as phagocytes and antigen-presenting cells. In AP, DCs infiltrate the pancreas and increase inflammation by secreting IL-6, TNF-α and MCP-1 (Ref. 61). Also, it has been described that acinar cells can transition to DCs and activate CD4+ T cells, thereby accentuating the local inflammation (Refs 57, 74). On the other hand, some studies have demonstrated the protective effect of DCs on the pancreas by inducing severe forms in mice depleted of DCs (Refs 57, 75).

#### NK cells

NK cells are a subtype of circulating lymphocyte that has a cytotoxic effect on tumoural and virus-infected cells. The cytotoxic effect is accomplished by inducing cell apoptosis by releasing perforins and lytic proteins from cytoplasmatic granules (Refs 54, 76). Besides this function, NKs are involved in inflammation through cytokine secretion (interferon-γ (IFN-γ), TNF, IL-5, IL-10 and IL-13) and chemokines (CCL3, CCL4 and IL-8) (Refs 57, 77).

NK cells regulate immune response by activating macrophages, DCs and T cells through the cytokine secreted and surface receptors. On the other hand, NK can kill these cells (DCs and macrophages) by cytotoxic effect (Ref. 76).

Regarding the involvement of NK in AP, evidence suggests that the number of NKs in the peripheral blood is decreased compared to the healthy individual. Moreover, in severe forms, there is an even more considerable decrease and this depletion is correlated to the infectious complications in AP (Ref. 57).

#### Inflammasomes

Inflammasomes are a cluster of multiprotein complexes found in the cytosol involved in the inflammatory response as part of innate immunity (Refs 78, 79). These complexes have two important components: one is the upstream sensor protein that belongs to the NLR (NOD like receptor) or ALR (AIM2 like receptor) family, and the other one is the adaptor protein, containing a caspase recruitment domain (ASC - apoptosis-associated speck-like protein containing a caspase activating and recruitment domain) (Ref. 78). The most investigated inflammasome from the NLR family is the NLR pyrin domain-containing protein 3 (NLRP3), which is expressed in monocytes, neutrophils, lymphocytes, DCs and epithelial cells (Ref. 78). NLRP3 is activated by both PAMPS and DAMPs. However, in AP, the recognition of DAMPs by the NLRP3 is the first step in its activation and afterwards in the induction of sterile inflammatory response (Ref. 78). Inflammasomes need two different signals for activation. The first signal requires the upregulation of inflammasome mRNA by NF-κB and the second signal begins the activation of pro-caspase-1 to its active form, caspase-1 (Refs 80, 81). There are also two pathways for NLRP3 inflammasome action. The canonical pathway activates caspase-1 and subsequently induces the maturation of IL-1β and IL-18, as well as pyroptosis, a form of programmed cell death discussed earlier. In contrast, noncanonical activation of caspase-1 requires the presence of caspase-11 in addition to the NLRP3 inflammasome (Ref. 45). NLRP3 inflammasome is involved in AP inflammation by activating IL-1β and IL-18. These two cytokines are secreted by activated macrophages but require activation from the precursor form, pro-IL-1β and pro-IL-18, to the mature activated form (IL-1β and IL-18). There are two steps in the activation process: the first one is the increased expression of inflammasome components and pro-IL-1β by the NF-κB transcription factor and the second one is the proteolytic maturation of the pro-molecules in the presence of NLRP3 inflammasome and caspase-1 (Ref. 82). The involvement of NLRP3 in AP pathogenesis is sustained by experimental studies, which showed that the absence of caspase-1, ASC or NLRP3 substantially reduced oedema and inflammation in a pancreatitis mice model (Refs 78, 83). Furthermore, there is also clinical evidence of the involvement of inflammasomes in AP. In a study conducted by Algaba-Chueca et al. (Ref. 84), the levels of NLRP3 and derived IL-1β and IL-18 were elevated in the first stages of AP. Another evidence of the importance of these molecules in AP comes from experimental studies that showed decreased severity of AP when NLRP3 inhibitor was used (Ref. 85).

### Adaptive immune response

**Figure 3.**
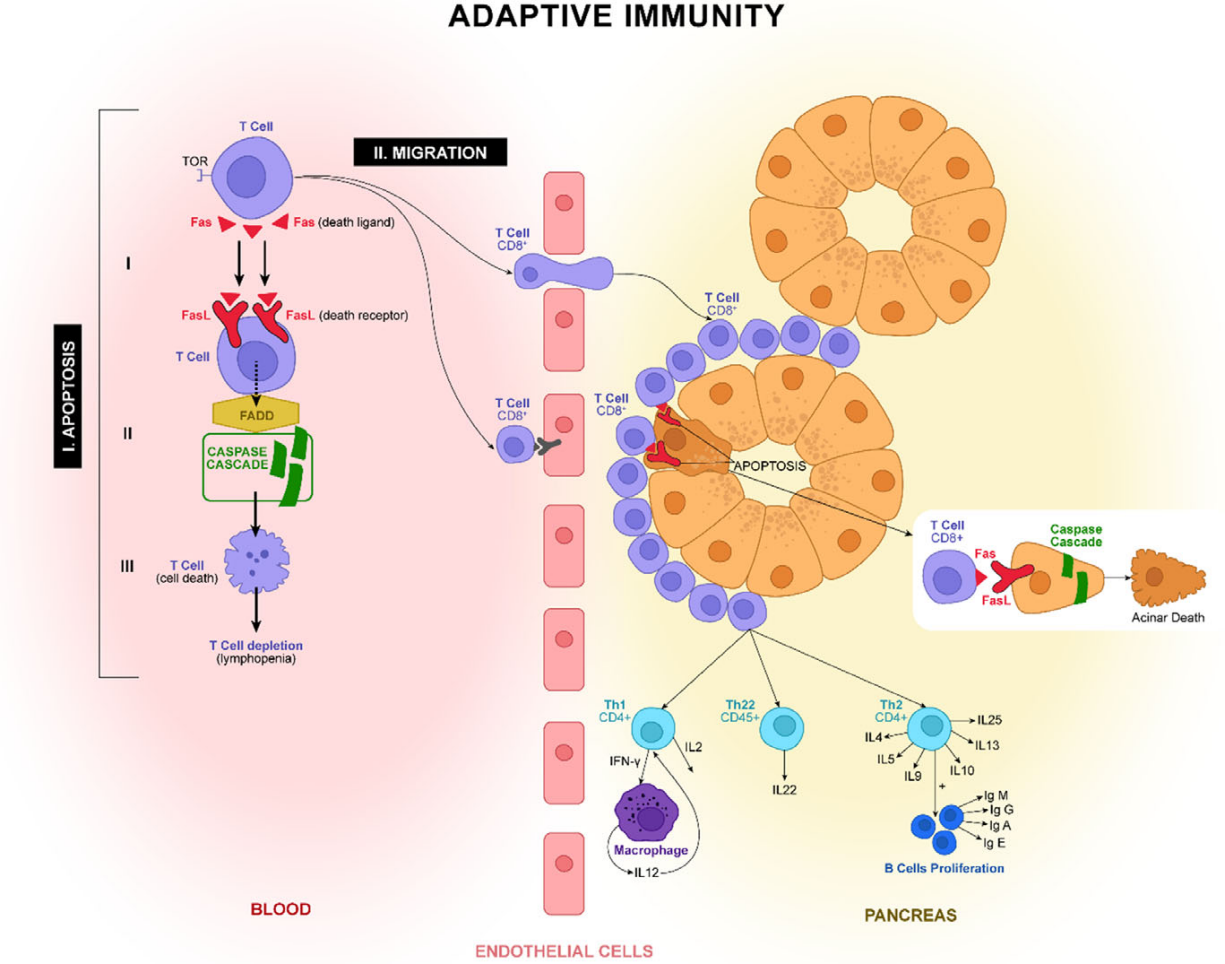
In acute pancreatitis (AP), blood T cells are reduced due to two mechanisms: the overexpression of Fas and FasL ligands on the surface, leading to premature apoptosis and migration to the inflamed pancreas. The T cells differentiate in the Th1, which augments the inflammatory response through proinflammatory cytokine production, and Th2, which secretes anti-inflammatory cytokines. The imbalance between these two types, with the predominance of Th1 response, is one of the most important factors for AP progression. Another important subtype of T cells is Th22, which reduces the inflammation in the pancreas by secreting interleukin 22 (IL-22). The role of B cells is not very well understood, but it seems they can play a bivalent role, pro- and anti-inflammatory.

An important role in triggering and shaping the immune response in AP belongs to adaptive immunity (acquired immunity). Adaptive immunity consists of the body’s response against self-antigens and non-self-antigens after presenting the antigenic particles via major histocompatibility complex (MHC) class I and II molecules to T cells. Repeated exposure to an antigen will activate both humoral immunity (antibody-mediated immunity mediated by B lymphocytes) and cellular immunity (mediated by T lymphocytes), and will trigger a hyperinflammatory process (Ref. 86). Damage of the acinar cells will determine pancreatic infiltration with innate immune cells (neutrophils and macrophages) and the abnormal activation of adaptive immune cells. In the more severe forms, they will trigger a systemic inflammatory response (SIRS) or MODS. Subsequently, T cells determine a compensatory anti-inflammatory response (CARS) (Ref. 44). Figure 3 show the main adaptive immune cell and mechanisms involved.

#### T cells

In the initial phases of pancreas injury, there is a reduction in the number of T cells (CD4 and CD8) in the circulation due to an excessive expression of Fas and FasL ligands on the cell surface. Fas (also known as CD25 or Apo-1) is a member of the TNF receptor family, which promotes lymphocyte apoptosis (Refs 87, 88). FasL (named CD95L or CD178) is a type-II transmembrane protein expressed on cytotoxic T lymphocytes and NK cells, which also induces programmed cell death (Ref. 89). This overexpression of Fas ligands results in a significant loss of peripheral T lymphocytes, immunosuppression and bacterial superinfection. The number of peripheral T cells is also reduced due to their migration to the inflamed pancreas (especially CD4 cells infiltrating the inflamed acini). Usually, the reduction is transient in moderate AP (MAP), with the number of cells normalizing after the fifth day of evolution. In the severe forms of AP (SAP), the severity of cellular destruction is correlated with persistent lymphopenia, especially with the reduction of Th cells, which amplifies the complication risk (Ref. 86).

Cytokines released secondary to the pancreas injury (IL-2, IL-4 and IL-12) will determine the differentiation of T cells in T helper (Th1, Th2, Th9, Th17, Th22 and follicular T helper) and regulatory T cells (Treg cells).

##### T helper cells

The Th1 and Th2 subtypes probably play the most important role in adaptive immunity. Th1 cells mainly produce proinflammatory cytokines to mediate cellular immunity, while Th2 cells produce anti-inflammatory cytokines to regulate humoral immunity (Ref. 90). In AP, Th1 cells participated in the early inflammatory response by releasing proinflammatory cytokines, while the Th2 subset secretes anti-inflammatory cytokines to counterbalance excessive inflammatory response (Ref. 91).

Th1 (CD4 cells) is essential in the adaptive immune system. Th1 increases cell-mediated response against intracellular bacteria by releasing different mediators (IL-12, IFN-γ and IL-2), activating macrophages, cytotoxic T cells and B lymphocytes. In addition to stimulating the cellular immune system, Th1 promotes the production of IgG, an opsonizing antibody (Ref. 92). IFN-γ stimulates macrophages to produce IL-12 and IL-12 stimulates the production of IFN-γ in Th1 via positive feedback. IFN-γ also inhibits the production of IL-4, an important cytokine that induces the differentiation of naive helper T cells (Th0 cells) to Th2 cells (Ref. 92).

Th2 leads to a humoral immune response by releasing different cytokines (IL-4, IL-5, IL-9, IL-10, IL-13 and IL-25), which stimulate B cells, Th1, eosinophils, basophils or mast cells. Th2 will stimulate B-cell proliferation and antibody production (IgG, IgM, IgA and IgE antibodies). The Th1/Th2 ratio value often correlates with the severity of pancreas injury. The predominant Th2 cells are a marker of favourable evolution and the mediators synthesized by these cells are found in large quantities in MAP. The decrease in the Th1/Th2 ratio correlates with an increased level of IL-6, TNF-α and IFN-γ, which secondary leads to reduced levels of IL-4 (Ref. 93). Reversed, in SAP, there is a discrepancy regarding the number of Th1, which persistently increases during the first days of evolution. Furthermore, the ratio of Th1/Th2 in SAP remains altered over time, being elevated even after 336 h (Ref. 94).

Th9 is a new subgroup of CD4 cells, which secretes IL-9, an important mediator of the enrichment of immune cells, such as eosinophils and mast cells (Ref. 95). Even though its role in inflammatory disorders or parasitosis is known, its involvement in AP etiopathogenesis has not yet been described.

Th17 is a unique subset of CD4 cells that plays a significant role in AP by producing IL-17, an important cytokine that protects the body against extracellular bacteria or fungi. After their activation by different cytokines, Th17 expresses IL-21, which is important in RORγt and IL-17 expression, determining T-cell proliferation and counteracting suppression by Tregs. Th17 also produces other inflammatory cytokines (TNF-α, IL-22 and IL-26), which play an important role in inflammation (Refs 96, 97). IL-22 induces antimicrobial proteins, defensins, acute-phase proteins, inflammatory cytokines and chemokines. Many studies have shown that IL-22 protects against AP, reducing acinar cell apoptosis, enhancing acinar cell regeneration and limiting inflammation or fibrosis (Ref. 98). IL-26 is a novel proinflammatory cytokine overexpressed in activated or transformed T cells. IL-26 stimulates the production of IL-1β, IL-6 and TNFα by human monocytes and is an essential antimicrobial agent, able to form pores in bacterial membranes (Ref. 99).

Th22 cells are a subpopulation of CD4 T cells implicated in the protective mechanisms against various bacterial and viral pathogens. Through their production of IL-22, these cells are involved in tissue repair processes and regulate innate immune mechanisms by producing chemokines and cytokines (Ref. 100). IL-22 has an important antimicrobial role, stimulates innate immunity, maintains tissue integrity and enhances tissue repair. Besides Th22, many other cells produce IL-22 in the body (Th1, Th17 cytotoxic T-cell subsets, γδT cells, NK and NKT cells) (Ref. 101). In SAP, the IL-22 produced by Th22 decreases as the disease progresses. It has also been proven that pulmonary complications in SAP are mediated by melatonin due to the upregulation of IL-22 and Th22 activities (Ref. 102). In 2016, Qiao et al. (Ref. 103) described the protective effect of recombinant IL-22 against L-arginine-induced SAP in mice by enhancing the expression of anti-apoptosis genes. Other studies that followed also described the beneficial role of IL-22, especially against the complications of SAP (Ref. 104). Elevated serum levels of IL-22 have been found in patients with AP, demonstrating the protective role of Th22 by interfering with autophagy processes. Th22 cells can stimulate the production of the anti-apoptotic factors (Bcl-2 and Bcl-XL), binding Beclin-1, an indispensable regulator of autophagy. Thus, Th22 blocks the process of acinar self-destruction, preventing pancreatic injury primarily via IL-22 (Ref. 105).

T follicular helper cells (Tfh) are a specialized subset of CD4+ T cells with a critical role in protective immunity by constantly helping B cells to produce antibodies. Tfh cells are located predominantly in secondary lymphoid organs (tonsils, spleen and lymph nodes) at the level of the B-cell zone, constantly interacting with B cells and favouring their maturation and differentiation (Ref. 106). Tfh cells recruited CD8+ T cells and B cells by secreting CXCL13 (chemokine ligand 13) and promoted the maturation of B cells into antibody-producing plasma cells by secreting IL-21. An important number of Tfh are found in the peripheral blood circulation, fulfilling similar roles to germinal ones and releasing many cytokines like (IL-4, IL-6, IL-5, IL-13, IL-17, IL-21 and IL-22) (Ref. 107). Tfh cells increased significantly in patients with AP and have a significant role in the development and progression of AP, primarily through the implication of IL-6 and IL-21 (Ref. 107).

##### Treg cells

Tregs are a specialized subpopulation that suppresses other immune cells and prevents the body from attacking itself, maintaining immune homeostasis and self-tolerance in this way. Tregs can inhibit T-cell proliferation and cytokine production, which is essential to protect against autoimmunity (Ref. 108). In AP, Treg activation determines the failure of the duodenal barrier function and favours commensal bacterial translocation into pancreatic necrosis (Ref. 109). Tregs also control proinflammatory response by reducing the activity of DC and NK cells, and promoting the differentiation of macrophages in those of type 2, with anti-inflammatory effects. However, in SAP, there is a substantial increase in the percentage of Tregs that is maintained as the pancreas destruction progresses. So, the number of Tregs is an important marker of unfavourable evolution in AP (Ref. 86). In AP, Tregs play a significant role in the counterbalance against SIRS syndrome. Infected pancreatic necrosis, a tardive complication of necrotizing AP, is secondary to bacterial translocation from the bowel associated with anti-inflammatory response syndrome (CARS) mediated by Tregs (Ref. 109).

##### Cytotoxic T lymphocytes (CD8 cells)

The role of CD8 cells in the adaptive immune system implies the responses of their T-cell receptors to diverse antigens presented by MHC class I molecules by proliferating, secreting cytokines and chemokines, and directly lysing infected cells (Ref. 110). CD8 cells become activated after they recognize the antigen and start the attack against infected cells in three directions. They secrete cytokines with antimicrobial effects (TNF-α and IFN-γ), produce and release cytotoxic granules (perforin and granzymes) and, probably the most important function, CD8 determines the destruction of infected cells via Fas/FasL interactions (Ref. 111). To date, there is no consensus about the implication of CD8 cells in AP. Some authors describe a significant depletion of these cells in SAP, others an increase in the CD8 level, while others reported no difference (Ref. 103, 112).

Cytotoxic T lymphocytes and NK cells have complementary roles. If the virus escapes the CD8 action, NK cells will intensify their action. As part of innate immunity, NK cells will recognize infected cells and determine cellular death by secretory lysosome exocytosis (Ref. 113).

#### B cells

B cells play an important immunomodulatory role. They secrete anti-inflammatory cytokines (IL-10, IL-35 and TGF-β) that inhibit the activation and proliferation of proinflammatory immune cells (Ref. 114). By producing IL-10, B cells block the immune response of Th1 and Th-17 cells, reducing their proinflammatory activity (Ref. 115). IL-35 is a recently discovered anti-inflammatory cytokine from the IL-12 family produced by a wide range of regulatory lymphocytes. It has an important role in blocking the development of Th1 and Th17 cells (Ref. 116). In a study conducted by Aksoy et al. (Ref. 117), the level of IL-35 was significantly lower in people with AP compared with healthy people, this marker being considered in the diagnosis and follow-up of patients with AP.

In the first stages of AP, there is an increased level of B cells, correlated with mild forms of disease. Reversely, in patients with SAP, a reduced number of circulating B cells, especially of regulatory B cells that produce IL-10, and a decreased level of immunoglobulins M and G (IgM and IgG) are often found. IgG levels seem to be an important prognostic marker, being very low in patients who developed infectious complications or those who died (Ref. 118). Furthermore, increased B-cell-activating factor (BAFF) levels were detected in patients with SAP. BAFF is a major cytokine that regulates B-cell survival, maturation and differentiation. BAFF acts as an acute-phase reactant and, like IL-6 or procalcitonin, can predict the severity of AP; these parameters were found to be significantly higher in patients who developed necrosis or who required intensive care hospitalization for more than 7 days (Ref. 119).

Studies conducted on mice proved that the value of amylase and the histological score of AP increases inversely proportional to the reduction in B lymphocytes. It is now established that B lymphocytes contribute to the severity of AP but the exact role of B subsets in PA etiopathogenesis still needs to be elucidated.

## Biomarkers for prognosis in acute pancreatitis

The first 48 h from onset are critical for identifying patients at risk for severe forms or complications. This timeline is also considered a period in which efficient measures can be taken to prevent severe complications and fatal outcomes (Ref. 120).

Given that, until now, no marker or scoring system has demonstrated a good predictive value in identifying the severe form, there is a continuous search for new molecules.

Based on the pathophysiological mechanisms involved in the progression of AP, several biomarkers could be helpful in the severity stratification; some already showed promising results and others have not yet been investigated. The most investigated markers have been the proinflammatory cytokines and chemokines like IL-6, IL-8, IL-1, IL-10 and IL-17. These molecules play an important role in the evolution of AP but are also unspecific markers of inflammation. There are fewer studies, but with promising results, regarding the more specific pancreatic markers like TAP, CAPAP and markers of initial cell migration and activation (PMN elastase, ICAM, MMP-9 and MCP-1) (Refs 120, 121).

- TAP is an amino-acid peptide with a low molecular weight rapidly excreted in urine. It is a cleavage product of trypsinogen, released into the systemic circulation with zymogen granule activation. TAP has been used as an early prognostic marker in AP, being significantly higher in patients with SAP versus MAP in the first hours of evolution (Ref. 122). In 2004, a study from the United Kingdom showed that the level of urinary TAP in patients admitted to hospital within 24 h of the onset of symptoms of AP could predict the disease’s unfavourable evolution. Urinary TAP measuring also proves helpful in the early evaluation of the AP severity (Ref. 123).
- CAPAP is a peptide released from procarboxypeptidase B and is a marker of the activation of pancreatic enzymes (Ref. 124). Both serum and urinary CAPAP can be considered early prognostic factors in predicting the severity of AP (Refs 125, 126). Muller et al. (Ref. 127) demonstrated that CAPAP levels have a predictive value of over 90% in identifying the development of pancreatic necrosis. CAPAP and TAP are both good prognostic markers, although TAP seems more accurate on the first day of admission (Ref. 128).
  These activation peptides of pancreatic proteases (TAP and CAPAP) are important in assessing the severity of AP at the beginning of the disease and they will probably be the basis of new studies to identify those patients who will require intensive care therapy (Ref. 129).
- MCP-1 is a chemokine involved in the attraction of monocytes to the inflammation site (Ref. 130). Yang et al. (Ref. 131) showed statistically significant differences in the values of MCP-1 at admission between the mild, moderate, and severe forms, making MCP-1 a good marker for risk stratification. Another study that sustained these findings found significant differences in MCP-1 concentrations between controls and AP patients, regardless of the severity (Ref. 132). A study conducted by Regner et al. (Ref. 133) demonstrated that both blood and urine levels of MCP-1 are higher on the first day of admission and decrease gradually in the following days. Serum MCP-1 levels seem superior in predicting SAP at admission compared to urinary levels (Refs 126, 133).
- ICAMs (ICAM1) are membrane glycoproteins that promote leukocyte migration to the site of inflammation. ICAM serum values are elevated in AP, especially in the severe or necrotizing forms (Refs 134–137). Zhu et al. (Ref. 138) established a cutoff value of 25 ng/ml for differentiating SAP from MAP with a sensibility and specificity of over 60%. The prognosis accuracy of ICAM was similar to the APACHE II score and superior to IL-6.
- MMP-9 is an endopeptidase involved in extracellular matrix degradation and, in AP, facilitates neutrophil migration in the pancreas. Neutrophils and macrophages secrete it. Several studies have demonstrated a significant increase in serum values of MMP-9 compared to mild forms or controls. MMP-9 could not discriminate between moderate and severe forms (Refs 139–141). In another study, MMP-9 showed a very good specificity but low sensibility in predicting SAP at admission (Ref. 142).
  Vitale et al. (Ref. 143) confirmed these findings in a small study in children with AP, showing significant differences in MMP-9 levels between mild and severe forms, making this a potential marker for paediatric patients, where the need for new markers is even more important.
- PMN elastase (neutrophil elastase) is an enzyme that results after neutrophil activation and degranulation. In AP, blood levels of elastase are increased. A few studies have shown a correlation between the blood level of elastase and the severity of the disease, proving the ability to serve as an early predicting marker. Domingues-Munos et al. (Ref. 144) demonstrated a good sensibility and specificity for detecting SAP in the first 48 h. Other studies found the same correlation but with a lower predictive value. PMN (polymorphonuclear neutrophils) elastase seems more accurate in predicting SAP at 48 h of admission than CRP (C-reactive protein) (Refs 145–147).
- TNF-α is a major cytokine involved in the inflammation from AP. TNF-α is secreted from various cells and molecules, from acinar cells to DAMPs, endothelial cells, neutrophils, macrophages and T cells (Ref. 148). TNF-α needs enzymatic conversion to its soluble form. Also, the activity is dependent on receptor binding. For these reasons, serum measurements can be difficult and few studies exist (Ref. 148). The results regarding the prognosis value of TNF-α are conflicting. One study found no connection between serum levels and severity of AP, whereas another study found higher levels of TNF-α in patients with gallstone AP compared to controls (Refs 149, 150).
- IL-8 is a chemokine secreted in AP by macrophages, monocytes, activated neutrophils and non-immune cells, like endothelial and epithelial cells, in response to cellular injury (Ref. 151). Several studies show the predictive value of IL-8 for severe forms (Refs 120, 152). One study demonstrated a sensibility of 60% and specificity of 80% in predicting progression to SAP (Ref. 153). Also, IL-8 levels correlate well with infected necrosis and systemic complication development (Ref. 154). To date, IL-8 is one of the best predicting markers of outcome but its clinical use is still limited (Ref. 153).
- IL-6 is produced by several immune cells alongside injured pancreatic cells and endothelial cells (Ref. 151). It is the main cytokine involved in the early phase of inflammation in AP and the most investigated cytokine as a marker for diagnosis and prognosis with promising results. IL-6 has shown good results in severity stratification and good predictive value for developing SAP (Refs 155, 156). It also correlates with distant organ failure, especially pulmonary involvement (Ref. 152). Compared with other markers or already used scores, IL-6 seems superior in predicting severe forms, with good sensitivity and specificity (Refs 155, 157, 158). However, clinical use is still limited due to rapidly decreased plasma levels and low accessibility (Ref. 151).
- IL-10 is an anti-inflammatory cytokine synthesized by the Th2 cells, B cells and monocytes. IL-10 decreases proinflammatory cytokine levels, especially IL-8 and MCP-1 (Ref. 151). There are conflicting results in the prognosis value of IL-6 in AP. Some studies showed increased serum levels in patients with AP compared to controls on the first day of admission. Also, patients with mild AP had higher levels of IL-10 compared to those in severe forms (Refs 159, 160). On the other hand, a study by Rao et al. (Ref. 153) found no association between IL-10 and severity.
- IL-17 is a proinflammatory cytokine secreted by Th17, γδ T cells and NK cells (Ref. 161). Recently, it has become a molecule of interest as a prognosis marker and a therapeutic target in AP (Ref. 162). Jia et al. (Ref. 163) demonstrated that IL-17 is a good marker for severity stratification if measured in the first 48 h of admission. Other studies showed a good predictive value not only for SAP but also for systemic complications and mortality (Refs 164, 165). The data available until now demonstrate a good prospective value of IL-17 as a single marker for identifying severe forms, but more studies are needed.
- IL-22 is a cytokine produced by Th22 and other T cells (Th1 and Th17). Regarding the IL-22 role as a marker of AP severity, opinions differ. Some authors sustained that the IL-22 level is not correlated with the severity of AP, while others demonstrated an important correlation between the IL-22 level and the severity degree of AP (Refs 166, 167). More studies are needed to understand better the role of this cytokine in the pathogenesis of AP and its predictive value.
- IL-21 has a broad immunomodulatory spectrum in innate and adaptive immunity, determining immune response suppression due to its inhibitory role on DC activation and promoting the proliferation of the immune cells by IL-10 and B-regulatory cell production (Ref. 168). A significant increase of IL-21 (promotor of inflammation) has been found in patients with SAP compared with those who developed medium forms of the disease. Still, the exact role of this cytokine in AP has not been established yet. It is supposed to produce immune paresis by inhibiting Treg cells (Refs 86, 169).
- IL-23 is an important cytokine from the IL-12 family. It has a role in stimulating Th17 cells, neutrophils and macrophages. Il-17/IL-23 plays an essential role in inflammatory diseases (Ref. 170). Jia et al. (Ref. 164) demonstrated significantly increased IL-23 levels in SAP patients compared to that in MAP patients and controls. The same study showed a positive correlation between IL-23, IL-17 and CRP in predicting severe forms.

## Conclusions

The pathogenesis of AP has long been investigated, but it still has not been fully understood. It is now clear that the immune system plays a pivotal role in this process. It starts from the local injury to the acinar cell that leads to intracellular changes like calcium overload, zymogen activation and eventually to a form of cellular death, and continues with the activation of innate and adaptive immunity and the systemic involvement of AP. The evolution of the disease is correlated with the degree of immune response and the imbalance between proinflammatory and anti-inflammatory molecules.

Understanding all the mechanisms and molecules involved in the development and progression of AP opens the way not only to possible new therapeutic targets but also for a better prediction of severity.

Based on these findings, new research should aim to find new, efficient and reliable markers for disease severity, as well as molecular targets for treatment.
